# Microelectronics-Based Biosensors Dedicated to the Detection of Neurotransmitters: A Review

**DOI:** 10.3390/s141017981

**Published:** 2014-09-26

**Authors:** Maryam Mirzaei, Mohamad Sawan

**Affiliations:** Polystim Neurotechnologies Laboratory, Electrical Engineering Department, Polytechnique Montreal, Montreal, QC H3T1J4, Canada; E-Mail: maryam.mirzaei@polymtl.ca

**Keywords:** microtechnology, microsystems, microelectronics, biosensors, chemical messengers, neurotransmitters

## Abstract

Dysregulation of neurotransmitters (NTs) in the human body are related to diseases such as Parkinson's and Alzheimer's. The mechanisms of several neurological disorders, such as epilepsy, have been linked to NTs. Because the number of diagnosed cases is increasing, the diagnosis and treatment of such diseases are important. To detect biomolecules including NTs, microtechnology, micro and nanoelectronics have become popular in the form of the miniaturization of medical and clinical devices. They offer high-performance features in terms of sensitivity, as well as low-background noise. In this paper, we review various devices and circuit techniques used for monitoring NTs *in vitro* and *in vivo* and compare various methods described in recent publications.

## 1. Introduction

Neurotransmitters (NTs) are the primary chemical messengers that are released from the neuron terminals upon depolarization. It has been shown that the mechanisms of several brain-level diseases are linked to NTs, such as Parkinson's disease and epilepsy. Regarding these two disorders alone, statistics show that over 300,000 Canadians and 50 million people worldwide have epilepsy [1], while approximately 100,000 Canadians have Parkinson's disease [2]. The concentration of some NTs in the human body is also related to diseases, such as Alzheimer's, with a drop in acetylcholine concentration [3], or Parkinson's, with a lack of dopamine (DA), as a result of the progressive degeneration of DA-generating cells in the substantia nigra, an NT that regulates movement [4]. The mechanisms of other diseases, such as epilepsy, are also linked to NTs, notably gamma-aminobutyric acid (GABA, the main inhibitory NT of the brain) and glutamate (GLU, the main excitatory NT of the brain). Because NTs have the potential to serve as clinically-relevant biomarkers for specific diseases and allow for treatment efficacy, their detection and characterisation are important. A sensor capable of continuously measuring NTs, such as DA, in the bloodstream or tissue *in vivo* would give clinicians a valuable window into patients' health and their response to therapeutics.

NTs are synthesized and stored in presynaptic terminals and have specific receptors on the postsynaptic cells. To detect and monitor NTs, various methods have been applied. Capillary electrophoresis (CE), microdialysis and liquid chromatography have been applied for the separation and fractionation of NTs, whereas laser-induced fluorescence, immunoassay and mass spectrometry (MS) have been used for their detection [5–7]. Because there are insufficient data regarding the origin of urinary NTs, studies have focused on measuring NTs *in vivo*. Additionally, it has been shown that neurotransmission occurs on the millisecond to minute time scale, which makes its real-time analysis easy to achieve [8,9]. Recently, the miniaturization of common chemical, biological and biotechnological manipulations and operations onto a chip (typically made by microfabrication) has given rise to a new concept called lab-on-a-chip (LOC). These LOCs include microfluidic structures, which have proven useful in the miniaturization of biological and analytical systems [10]. Microfluidics can be integrated for *in vivo* analyses that monitor and measure proteins and molecules, such as NTs. The microfluidic structure can be integrated using other techniques, such as microdialysis, to minimize analysis times and incorporate online sampling [11]. As an example, microdialysis is an effective tissue sampling strategy that has been applied to study many areas of the brain. However, it has not yet been assayed in real time; therefore, the method does not provide information on changes in neurotransmitter levels on the millisecond to second timescale. Several groups have worked to integrate microfluidics into microdialysis probes [12–14], enabling faster analysis times and the ability to sample at lower flow rates, resulting in higher sample recovery and greater temporal resolution. However, microdialysis has very low temporal resolution, and due to its large size, it is not suitable for implantable sensors [15]. Other studies in this area still do not fulfill the criteria that are important for implantable devices, for example small size and compactness, and they are also too bulky to be implanted [16].

Microsystems based platforms are popular in biological and clinical studies. They are miniaturized devices offering high-performance features in terms of sensitivity. They have low background noise, use cost-effective components and are able to perform measurements in turbid samples [16]. In biological processing, miniaturized systems are used in various applications, such as glucose sensors [17]. Though, as electrochemical biosensors were introduced in 1974 [18], scientists have been interested in developing these biosensors in various areas, because they are small volume, fast and parallelizable. Most biomolecules have extra electrical charges or can be modified with redox-active tags. It is possible to apply simple electrochemical detection schemes by using pre-defined electrical excitations to monitor molecular responses [19]. In particular, the electrochemical sensor is a completely label-free detection platform, which is beneficial to portable sensors [20].

Adams's laboratory was the first group to develop electrochemical methods for the detection of NTs [21]. Since then, electrochemical detection has been extensively used for monitoring NTs [22], because of its high sensitivity and fast response times [23–25]. Several NTs, such as DA, norepinephrine (NE), serotonin (5-HT) and nitric oxide, are electroactive and can be detected via electrochemical detection [26]. Using label-free detection, the analyte can be determined directly with very little or no sample preparation. In general, most electrochemical sensors for NTs do not integrate specific molecular tags, and they offer a compact and potentially label-free detection solution. They are sensitive to the offset and background noise and are normally subjected to noise in the reversible hybridization processes, resulting in a sensitivity of around 100 nM for molecular detection, which is much higher than the concentrations in regular biochemical experiments [27].In addition, nonspecific binding causes interference, making no discrimination between the signals of specific and non-specific interactions [28].

Different techniques can be applied to increase the sensitivity, which will be discussed further. Additionally, electrical noise could be improved by various techniques, such as additional post-processing steps, which can be applied to the employed metallic electrodes, or polymers to facilitate probe molecule deposition.

In this paper, we review the microelectronic techniques used for monitoring NT detection *in vitro* and *in vivo*. Different types of voltammetry, various categories of electrodes and coatings, CMOS technology and dielectrophoresis will be discussed. In addition, we will compare and evaluate methods described in publications within the past three years, 2010–2013, in terms of the type of method, the detected analyte, the limit of detection and whether it is *in vitro* or *in vivo*.

## 2. Voltammetry, Amperometry and Potentiostat

Voltammetry is an electrochemical sensing method that has high temporal and spatial resolution and excellent sensitivity, which is suited for studying the dynamics of intercellular communication and is extensively used for *in vivo* and *in vitro* measurements of NTs [29]. This electrochemical method directly detects NTs in the brain. In voltammetry, a low voltage (1–2 V) is applied across an electrode interface in solution, and the current developed via chemical reactions occurring at the electrode surface is monitored. Bi-directional charge transfer occurs between an electrode and an analyte in solid or liquid phase, which uses the applied electrode potential and provides energy for electron transfer (*i.e.*, a chemical reaction) to occur. The chemical reactions occur at the electrode's surface or at very short distances away within the probed sample volume, and the resulting charge transfer or current, which is proportional to the concentration of the electroactive NTs, are measured.

On the other hand, amperometry and fast-scan cyclic voltammetry (FSCV) are the two most commonly used voltammetric methods of NT measurement. Amperometric transduction is a current-measuring sensor with two electrodes, which is controlled at a constant potential and is highly sensitive to the concentration of an analyte. It is an accurate electrochemical sensing method commonly used for the detection of analytes after separations; however, it has low chemical selectivity, so many compounds can be detected at potentials that are sufficient for biogenic amine oxidization. In addition, constant-potential amperometry requires a standard potentiostat and is a straightforward technique for the collection and analysis of current signals generated at a constant applied potential [30]. Amperometry is also the method chosen for the detection of NT levels when the major electrooxidizable species are known and electroactive interferents are either selectively excluded from the sensing electrode surface or below the detection limit [26]. Alternatively, FSCV measures the analyte every 100 ms. It has been shown that FSCV offers increased specificity, because a chemical signature called a cyclic voltammogram is recorded to identify the detected species, and therefore, it is recognized as one of the best choices for the detection of endogenous NTs in animals [31]. FSCV can be an excellent electrochemical detection method for electroactive NTs, such as DA; however, it requires specialized instrumentation. In addition, FSCV offers scan rates greater than 100V/s and can be suitable for monitoring and quantifying DA or other redox-active bio-analytes with rapid changes in various concentrations [19].

The detection and quantification of NTs via voltammetry are mainly performed using carbon fiber microelectrodes (CFMs). These microelectrodes are commercially available with small-diameter carbon fibers, and the fabrication methods are accessible. Carbon-based electrodes have been used extensively in voltammetric analysis, because they offer low-cost, chemical stability and wide potential and electrocatalytic activity ranges for specific redox reactions. They are also more biocompatible than metal electrodes, making them more appropriate for application in biologically-relevant redox systems and *in vivo* analysis [32]. However, these electrodes have poor chemical selectivity, and fouling occurs during the test [29]. Electrode fouling is caused by the macromolecules in the brain, as well as the slow electron-transfer kinetics at the electrode surface, resulting in low-sensitivity electrochemical detection. Bio-fouling also occurs because of the blockage of the electrode surface over time by matrix components, such as proteins. Thus, bare electrodes cannot provide stable and reproducible data, due to the surface fouling created by the adsorption of oxidized products of some compounds on the electrode surface [33].

In order to enhance electrode sensitivity, electrode surfaces could be modified using polymer coatings, especially in neuroscience applications. The modification of electrodes with semipermeable membrane (permselective) polymers enhances the sensitivity and selectively of cationic NT detection [30,34]. Various approaches have been developed to modify electrodes using polymer film, nanomaterials, covalent modification and self-assembled monolayers, as well as the use of carbon paste electrodes and the electrochemical pre-treatment of the electrode [35]. Polymer-modified electrodes prepared via electropolymerization have also been applied for the detection of analytes. Selectivity, sensitivity, homogeneity, strong adherence to the electrode surface and excellent chemical stability are some characteristic of the film. As an example, for electrostatic improvement and the sensitive electroanalysis of cationic DA in a neutral solution, Su *et al.* reported on the thiol-ene chemistry-guided preparation of novel thiolated polymeric nanocomposite films of abundant anionic carboxylic groups [36].

The measurement by the modified electrode is linear with DA concentration, yielding good anti-interferent ability and stability. It has also been shown that all proteins do not behave similarly during electroanalytical measurements, and thus, careful consideration of the matrix effect is required during biological monitoring. For example, 5-HT, as an oxidative by-product, reduces electrode sensitivity, due to electrode fouling over time. Fagan-Murphy *et al.* examined the effect of different biological matrices in terms of 5-HT response on two commonly used carbon-based electrodes [37]. They suggested that all biological matrices cannot be treated in a similar way, even if the biological elements within a given matrix are potentially similar.

In another study, it has been shown that the surfactant, sodium dodecyl sulfate (SDS), in a paste (SDS-CP) electrode distinguishes between the cationic form of DA and the anionic electroactive species that exist in biological fluids at the physiological pH. Pătraşcu *et al.* reported on a modified carbon paste electrode that incorporates the anionic surfactant sodium dodecyl sulfate (SDS) in the paste (SDS-CP) [38]. The detection limit was achieved in the submicromolar range, and the technique has the ability to remove the interference of ascorbic acid (AA) and to reduce the interference of uric acid (UA). Ensafi *et al.* reported on a polymer film of Tiron that could be used to modify glassy carbon electrode (GCE) and showed the electrochemical behavior of AA, DA and UA at the surface of the modified electrode [39]. Various electrocatalytic activities of the modified electrode toward these species were achieved. The results showed that the later technique was comparable with the reported methods.

**Figure 1.**
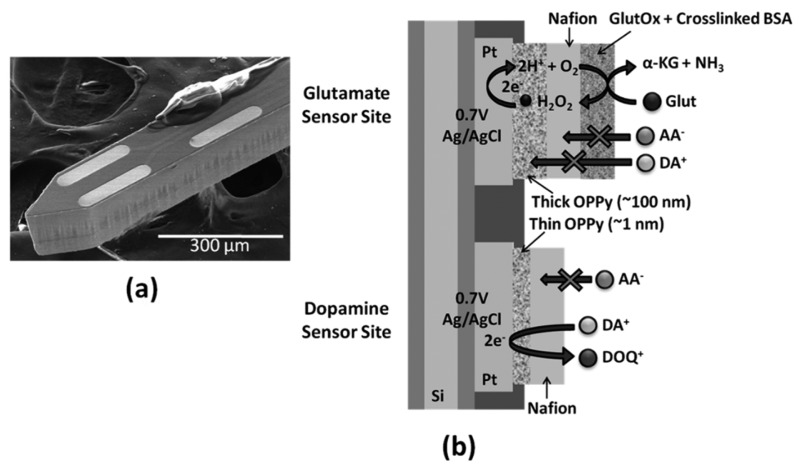
(**a**) Scanning electron microscopy (SEM) image of selective GlutOx immobilization on the top left microelectrode site previously modified with a thick overoxidized polypyrrole(OPPy) film and Nafion; (**b**) schematic diagram of the final dual glutamate (GLU)/dopamine (DA) sensor configuration. Reproduced with permission from [30].

To increase sensor selectivity against interferences, the electrode surfaces can be modified with Nafion to exclude anionic interferents, such as perfluorinated ionomer. In addition, overoxidized polypyrrole (OPPy) has recently been deposited on electrodes as a permselective cation-exchange film. Polypyrrole (PPy) is a conducting polymer that has been used in the design of bioanalytical sensors. Tseng *et al.* described an implantable microprobe with OPPy/Nafion-modified Pt microelectrode array microsensors for the combined, near-real-time monitoring of non-electroactive GLU and electroactive DA with high sensitivity and selectivity, as well as an adequate detection limit (Figure 1) [30].

In another study, Dengler *et al.* used a molecularly-imprinted polymer (MIP) electropolymer of OPPy for the *in vivo* detection of DA [40]. The MIP is formed in the presence of a molecule that is then extracted, leaving behind a complementary cavity with a chemical affinity for the original molecule.

**Figure 2.**
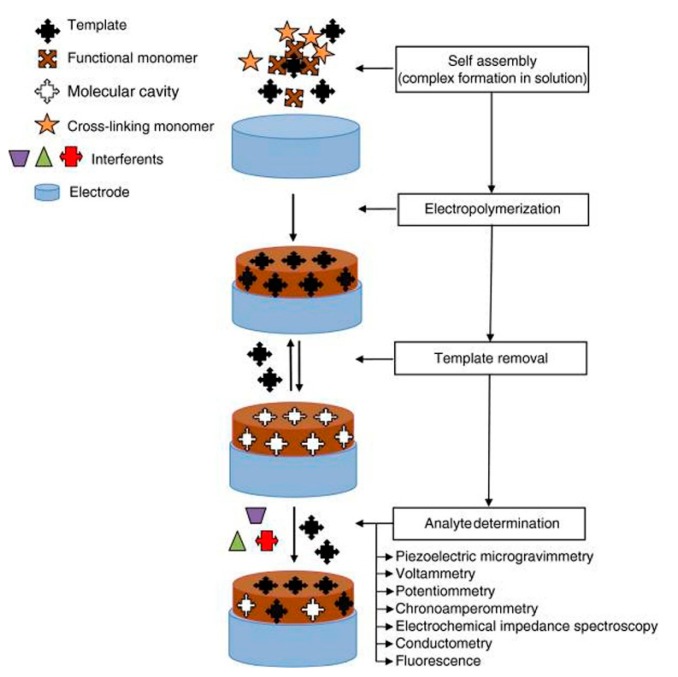
General procedure for molecular imprinting with an electroactive functional monomer and the typical signal transduction methods employed in detection. Reproduced with permission from [41].

Different polymers, such as PPy, poly(1,2-phenylenediamine), polyphenol and polythiophenol, are electroactive functional monomers that have been used widely for the development of chemosensors based on MIP [41]. Figure 2 shows the general procedure for MIP.

The functional monomer of pyrrole plays an important role in the MIP in terms of recognizing the desired DA target molecules. Zachek *et al.* presented the first FSCV microelectrode arrays (MEA) implemented *in vivo* with the ability to sample from various regions in close spatial proximity (equidistant within 1 mm), providing information about neurotransmission across multiple brain compartments [42]. The mentioned methods could modify electrodes and increase the sensitivity of the device to monitor NTs in a more reliable manner.

### 2.1. Voltammetry in Wireless and Implantable Devices

**Figure 3.**
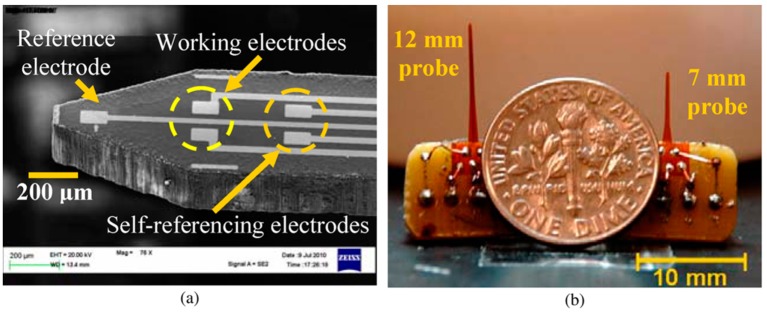
(**a**) SEM photo of the probe tip and (**b**) a photo of the assembled devices. Reproduced with permission from [44].

Voltammetry can be integrated into wireless devices, which makes it a good choice for implantable devices. Clark *et al.* described a biocompatible implantable voltammetric microsensor that can be applied to the targets of midbrain DA systems, detecting sub-second DA dynamics with sub-second temporal resolution over months *in vivo* in rats and mice [43]. It has been shown that FSCV offers the chemical selectivity needed to differentiate DA from other electroactive species in the brain by providing an electrochemical signature (cyclic voltammogram) of the analyte. The system has the ability to obtain multiple repeated recordings. In another study, Cao *et al.* developed an integrated flexible implantable probe on a polyimide-film substrate for sensing NTs. The flexibility of the probe helps to prevent scar formation in tissues during long-term *in vivo* monitoring. The assembled sensors were calibrated and tested at various concentrations of L-GLU with and without the presence of interfering molecules. The results demonstrated good sensitivity and selectivity (Figure 3) [44].

**Figure 4.**
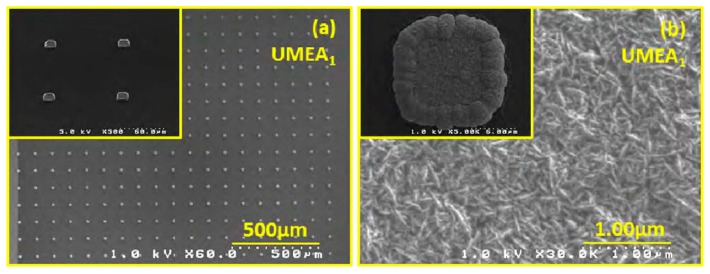
(**a**) Low magnification SEM micrograph showing a section of the ND-UMEA (ultra-microelectrode array) in a SiO_2_ matrix; inset: tilt view at 45° of the ND-UMEA projecting above the surrounding SiO_2_ plane;(**b**) High resolution SEM micrograph of the microstructure of the nanodiamond film; the inset shows an individual ND-UME with a “donut”-like geometry. Reproduced with permission from [19].

The wireless instantaneous NT concentration system (WINCS) also provides real-time co-monitoring of NTs. Shon *et al.* reported on the use of WINCS-based FSCV for the wireless, real-time, spatially and chemically resolved monitoring of adenosine (AD) at a carbon fiber microelectrode (CFM). It has been shown that the WINCS supports FSCV at a fiber CFM to provide superior temporal and spatial resolution, including chemical specificity [45]. The WINCS provided reliable, high-fidelity FSCV measurements of extracellular concentrations of AD, both *in vitro* and *in vivo*. These results demonstrate that WINCS is well-suited to the *in vivo* monitoring of AD, and the clinical application of AD measurements may prove important in achieving a better understanding of neurochemical mechanisms. In another study, Kang *et al.* used ultra-microelectrode arrays (UMEAs) (Figure 4), a square array of 50 × 50 microelectrodes, to detect DA [19].

UMEAs offer several advantages over macroelectrodes, because of having micron-sized electrodes, which enhance the rates of mass transport of the analyte to the electrode surfaces. The compatibility of microfabricated nanodiamond UMEA with the FSCV technique was evaluated, which was found to offer high temporal resolution for NT detection. Because DA is an electrochemically active compound and forms DA-ortho-quinone (DOQ), via oxidation, transferring to electrons and protons, the behavioral and environmental events of DA release occur on a short timescale. Dengler *et al.* found that electroactive species, such as DA, can be adsorbed to an electrode and then rapidly released by varying the potential of that electrode [40]. The desorbed species, in this case DOQ, causes a change in the local concentration that can be measured via an independent microelectrode in close proximity using FSCV. This technique could be applied to create an electrochemical sensor for measuring the basal tone or tonic concentrations of an electroactive biochemical in a tissue in real time. The application of voltammetry in implantable and wireless devices is promising for NT monitoring.

### 2.2. Voltammetry for Simultaneous Measurement of NTs

The simultaneous measurement of NTs is especially important in plasma and urine samples. Normally, AA, DA and UA coexist in body fluids. Because the oxidation potentials of AA, DA and UA are very close to one another, their selective detection using bare electrodes is a challenging task. Additionally, due to the surface fouling of bare electrodes caused by the adsorption of oxidized products of AA on the electrode surface, stability and reproducibility cannot be achieved. At bare electrodes, the selective detection of some NTs, such as DA, is impossible, because of interference from other compounds, such as AA and UA, which have oxidation potentials that are very close to that of DA [46]. To monitor AA, DA and UA simultaneously, conventional solid electrodes undergo an overlapping oxidation potential, and electrode fouling occurs because of the adsorption of oxidation products. To prevent this, various polymer films, nanoparticles (NPs), self-assembled monolayers and metal-oxide-modified electrodes have been used. Atta *et al.* used a composite-modified platinum (Pt) electrode for the simultaneous determination of catecholamines, UA and AA. Atta *et al.* also constructed an electrochemical sensor based on gold NPs and graphite for the selective detection of DA [47]. The gold NPs on the electrode surface amplified the signal considerably. Furthermore, the sensor showed sensitive and selective determination of DA in the presence of AA and UA. In another study, Wan *et al.* showed that an electrode modified by a poly(3-thiophenemalonic acid) film of a negatively-charged compound could work as an electrocatalyst for DA and UA oxidation and as a discriminating layer for DA against AA and UA; and that it could be used for the simultaneous determination of these compounds. In addition, using this modified electrode, the oxidation current of DA and UA increased significantly, as did the reproducibility and stability. The proposed methods can be applied to the detection of DA in human urine samples [48].

To monitor DA in the presence of AA and UA, Ni *et al.* developed a method to study the electrochemical behaviour of AA, DA and NA using differential pulse stripping voltammetry (DPSV) [49]. This method is simple, fast and cost effective for the simultaneous detection of NTs. Differential pulse voltammetry (DPV) is also used to make electrochemical measurements and is considered a derivative of linear sweep voltammetry or staircase voltammetry, with a series of fixed voltage pulses superimposed on the potential linear sweep or stair steps. The current can be measured directly before each potential change, and the difference of the current is plotted as a function of the potential. It was concluded that the chemometric methods of analysis would have to be applied to resolve the complex voltammetric outputs resulting from the mixture of the three analytes. In another study, Singh *et al.* compared three key coating materials to show their selectivity and fouling resistance to electrodes: Nafion, base hydrolyzed cellulose acetate (BCA) and fibronectin [29]. They found that BCA is relatively fouling resistant. Fibronectin coating creates moderate losses in sensitivity after coating and fouling. Nafion increases sensitivity for DA and norepinephrine, but not 5-HT. In addition, it has selectivity for cationic NTs over anionic metabolites. Even though Nafion resists fouling, both the dip-coating and electrodeposition of Nafion result in fouling that is similar to levels observed at bare electrodes after exposure to brain tissue. For the simultaneous determination of AA, DA and UA, Ensafi *et al.* used Sulfonazo III-modified film on a glassy carbon (GC) electrode. Sulfonazo III, a 2,7-bis(2-sulfophenylazo) chromotropic acid tetra sodium salt, is a pH indicator [39]. Thus, the electrochemical behavior of the modified electrode depends on the PH of the solution. They showed that the modified electrode has electrocatalytic activity toward the oxidation of AA, DA and UA. Wan *et al.* also presented the preliminary results of the simultaneous determination of DA and UA in the presence of AA by using a poly(3-thiophenemalonic acid)-modified glassy carbon electrode (3-TPA/GC) [35]. They showed that a poly(3-thiophenemalonic acid) film of negatively-charged compounds that formed on an electrode surface could act both as an electrocatalyst for DA and UA oxidation and as a discriminating layer for DA against AA and UA. Table 1 summarizes recent voltammetric techniques for the detection of NTs.

Overall, one of the most important aspects of voltammetry is the low noise and high sensitivity in biosensor applications, enabling the simultaneous detection of multiple analytes. Although voltammetry is one of the sensitive electrochemical analyses, expensive equipment and complicated procedures are required. More work needs to be done in this area to design simple and affordable devices for *in vitro* and *in vivo* applications. In the following section, we will further discuss carbon nanotubes, one of the most used modified electrodes in NT measurement.

## 3. Carbon Nanotubes Electrodes

Carbon nanotubes (CNTs) were first discovered by Sumio Iijima in 1991 [70]. Since then, studies have pursued their potential applications in various areas. CNTs have two structural forms, single-walled carbon nanotubes (SWNTs) and multi-walled nanotubes (MWNTs), both of which have applications in many areas, such as composite materials, nanoelectronics, energy research and biomedicine. CNTs have optical and electrical properties suitable for biomolecule detection. SWNTs have semiconducting behaviors, as well as resonance Raman scattering, making them suitable for use as nano-probes in biosensors. SWNTs can act as transistors; in this case, the molecules that bind to the surface of nanotubes act as the gating molecules and change the conductance of semi-conducting SWNTs. MWNTs grown on a platinum substrate were used as an amperometric biosensor.

**Figure 5.**
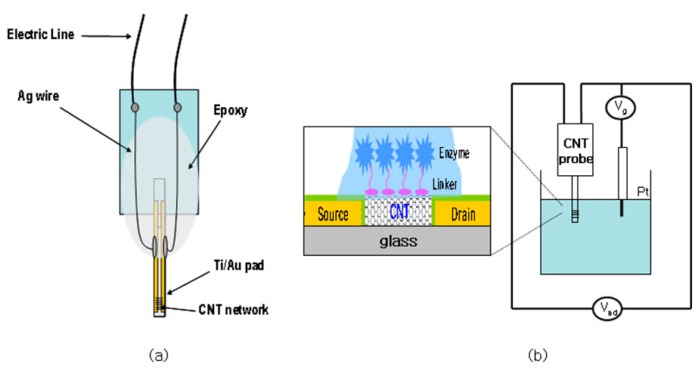
(**a**) Schematic structure of a probe-type CNT transistor; (**b**) schematic diagram of the experimental setup for sensing of a CNT transistor in aqueous solution. Reproduced with permission from [72].

Recently, several groups have applied CNT-based electrochemical biosensors for the monitoring of diverse biological structures, such as DNA, viruses, antigens and disease markers. These molecules can promote electron transfer in electrochemical reactions. The carbon nanotube coating on the electrodes is biocompatible and can be used *in vivo*, as well as *in vitro* [71]. Carbon-based electrodes are biocompatible and low-cost and have good electron transfer kinetics. CNTs can also been integrated into electrochemical sensors. They offer enhanced electronic properties and rapid electrode kinetics. Thus, CNT-based sensors normally have higher sensitivities, faster electron transfer kinetics and lower limits of detection than traditional carbon electrodes. CNTs also offer high electrical conductivity, mechanical strength and natural stability. CNT-based sensors can be used in common electrochemical methods, such as voltammetry, amperometry, electrical impedance spectroscopy and potentiometry. They can also be used as the gate in field-effect transistor (FET) sensors, because of high sensitivity to local chemical environments (Figure 5) [72].

To expand the array of analytes beyond electroactive molecules, enzymes are often incorporated into biosensors to selectively detect an analyte and create an electroactive product after an enzymatic reaction. Biomolecules, such as proteins, enzymes and DNA are easily adsorbed on to the surface of CNTs and can be attached directly to functional groups on the CNTs [22]. Adding CNTs to electrodes for NT detection could facilitate higher sensitivity and develop the electron transfer kinetics. The most direct electrochemical detection using CNT-based electrodes has focused on monoamines, including DA, 5-HT, NE and epinephrine (EP).

Dip coating CNTs is not reproducible, and sometimes, CNTs lay flat on the surface of the electrode, resulting in limited access to the electroactive sites. Xiao *et al.* introduced a new method for coating CNTs on the electrodes [73]. They aligned CNT forests using a chemical self-assembly method, resulting in more CNT ends exposed to the analyte. The modified electrodes were sensitive and had high temporal resolution, which would facilitate the electrochemical detection of NT release *in vivo*. Gholizadeh *et al.* also reported a sensitive GLU biosensor based on GLU dehydrogenase/vertically-aligned CNTs (GLDH, VACNTs). The results showed that the GLDH/VACNT electrode was a suitable basic electrode for the detection of GLU, even without an electron mediator. They also reported the fabrication of a vertically-aligned carbon nanotube nanoelectrode array (VACNT-NEA) via the photolithography method [74]. The fabricated VACNT-NEA and high-density VACNTs were used as electrochemical GLU biosensors. GLU dehydrogenase is covalently attached to the CNTs. The results prove the efficacy of the fabricated NEA as a low-cost and high-sensitivity enzymatic biosensor that is well-suited for the voltammetric detection of a wide range of clinically important biomarkers.

In another study, Jacobs *et al.* modified carbon-fiber microelectrodes (CFMEs) with three differently functionalized single-wall carbon nanotubes and measured their responses to 5-HT, DA and AA using FSCV. Both carboxylic-acid-functionalized and amide-functionalized CNTs increased the oxidative current of CFMEs by approximately 2–6-fold for the cationic NTs, 5-HT and DA, but octadecylamine-functionalized CNTs resulted in no significant signal change. PPy, as a conducting polymer, can also be used in the design of bioanalytical sensors [75]. Cesarino *et al.* developed a biosensor by using a simple electrocodeposition of MWCNT, PPy and Laccase (Lac) on a platinum (Pt) electrode surface [24]. The NT biosensor was applied to detect DA in urine samples using DPV. The Pt/MWCNT/PPy/Lac biosensor exhibited a detection limit of 0.14 mM, which was an adequate level for monitoring DA in urine, and the obtained results were in full agreement with those from the HPLC procedure. Cesarino *et al.* has also developed a new biosensor by a simple electrocodeposition of multi-walled carbon nanotubes (MWCNT), PPy and Lac on the platinum (Pt) electrode surface. The NT biosensor was applied to the detection of DA in urine samples using DPV [24]. Peairs *et al.* proposed using oPPY-CNT electrodes instead of Nafion-CNT electrodes for repeated *in vivo* use, because of their reproducible fabrication, selectivity over anions and larger currents *in vivo* [34]. Immobilizing CNTs into these polymers should further improve sensitivity by increasing the electroactive surface area. The selectivity for DA over AA was better with oPPY-CNT electrodes. The oPPY-CNT electrodes also displayed increased electron transfer kinetics for anions, while Nafion-CNT electrodes did not, proving that polymer deposition can affect the electrocatalytic properties of the CNTs. Although Nafion-CNT electrodes were easier to make, oPPY-CNT electrodes were more beneficial for DA detection, because they were reproducibly fabricated, measured higher currents *in vivo* and preserved selectivity over anions.

### 3.1. CNTs in Simultaneous Detection of Neurotransmitters

The simultaneous determination of NTs could be facilitated by using CNT. Njagi *et al.* developed implantable carbon microelectrodes that were modified with CNTs dispersed in nafion to quantify pharmacological alterations in 5-HT release via DPV [76]. Goyal *et al.* also reported the simultaneous determination of catecholamines EP and NE using square wave voltammetry (SWV) and a multi-walled carbon-nanotube-modified edge plane pyrolytic graphite electrode (MWNT/EPPGE) [77]. SWV has also been applied in NT measurement; it has a high sensitivity, suppresses background current and is a multipurpose technique for electroanalytical purposes. In another study, the same group used an edge plane pyrolytic graphite electrode modified with MWNTs/EPPGE for the quantitative detection of EP in the body fluids of smokers and non-smokers, using cyclic voltammetry (CV) and SWV. In this case, the voltammetric method was used to analyze EP concentration in plasma samples for the first time [78]. Generally, in a natural environment, EP often co-exists with high concentrations of other electroactive biomolecules, such as UA, DA, NE and AA, which interfere with one another. In addition, the product of EP oxidation (EPchrome) can easily be converted into polymers, blocking its further oxidation on the electrode surface. The UA and AA interference was overcome by applying a specific potential region, using MWNT/EPPGE for the determination of catecholamines and, thus, preventing UA and AA from interfering with the determination. Kamyabi *et al.* similarly reported the electrochemical behavior of AA, DA and UA on the surface of the iron (II) complex-MWNT-modified GC electrode [79]. The modified electrode showed sensitivity, selectivity and anti-fouling properties. They concluded that simultaneous or independent measurements of the three analytes are possible without any interference.

A self-assembled monolayer (SAM) can also be used for the modification of electrode surfaces, providing fast responses, high sensitivity, antifouling effects and chemical specificity. Pătraşcu *et al.* used a novel captopril/thiophenol (Capt/TP) mixed SAM as a substrate for the selective adsorption of 5-HT in a solution matrix containing AA and the other commonly encountered NTs. As a result, the charge-transfer impedances underwent large changes caused by very small changes in the electrode surface due to adsorption, and these changes were detectable [38]. Babaei *et al.* demonstrated that the simultaneous determination of adrenalin (AD) and paracetamol (PAR) can be performed on a single-walled carbon nanotube/chitosan/ionic-liquid-modified glassy carbon electrode (SWCNT-CHIT-IL/GCE). They showed that a biocompatible polymeric matrix, chitosan (CHIT), displays excellent film-forming abilities, high-water permeability and susceptibility to chemical modifications, and chitosan chains can associate with CNTs to form a biocompatible nanotube aqueous solution [80].

### 3.2. CNTs in Implantable Devices

**Figure 6.**
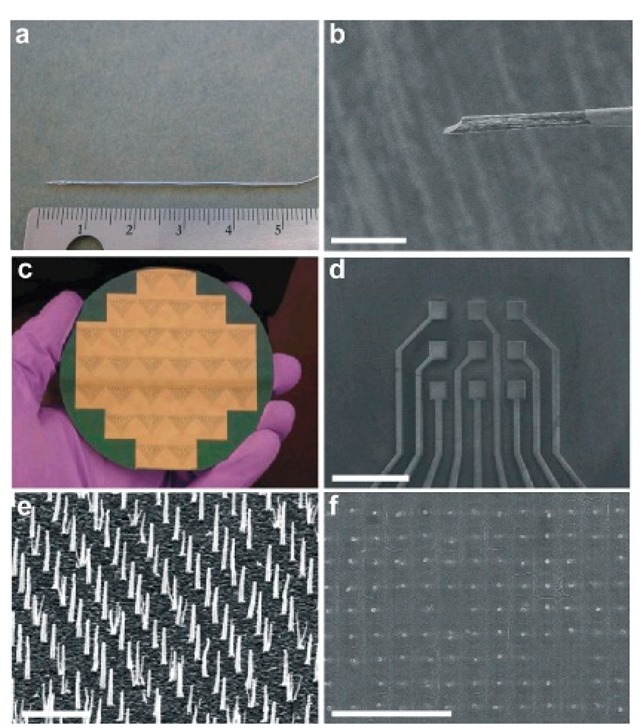
Images of CFM and CNF electrodes. Photographs of (**a**) CFM and (**c**) CNF devices after fabrication. Scanning electron microscopy images of (**b**) CFM encased in a borosilicate capillary and a (**d**) CNF-based 3 × 3 sensor pad array. High-resolution scanning electron microscopy images of (**e**) CNFs on a sensor pad prior to dielectric encapsulation and (**f**) CNFs on a sensor pad after dielectric encapsulation. Scale bars are 20 mm, 800 mm, 2 mm and 5 mm, respectively. An imposed tilt of 30°was utilized in (e). Reproduced with permission from [81].

The addition of CNTs to polymer coatings facilitates the measurement of small concentrations of NTs *in vivo*. Koehne *et al.* integrated a carbon nanofiber (CNF) (Figure 6) electrode array with the WINCS for the detection of DA using FSCV [81].

CNFs have been shown to be useful as neurochemical recording electrodes, because of their high sensitivity, fast electron transfer kinetics, widespread potential window, biocompatibility and manufacturing versatility. Additionally, FSCV is a particularly powerful technique that can be used to monitor very rapid events, such as neurochemical release. Due to their small size, both micro- and nano-electrodes exhibit a double-layer capacitance, which aids in the rapid change of electrode potentials and eliminates the distortions in the applied voltage waveform that are observed with larger electrodes. WINCS [82], which was developed at the Mayo Clinic, has been used in conjunction with a CFM to detect DA, GLU, AD and 5-HT *in vitro* and *in vivo*. Overall, CNTs could facilitate higher sensitivity and develop electron transfer kinetics for NT detection.

Although numerous encouraging results using CNTs in biomedicine have been published in the past several years, more work is yet needed before CNTs can enter the clinic. One of the important issues for CNT application is the concern of long-term toxicity. It has been shown that well-functionalized carbon nanotubes are not toxic *in vitro* to cells and *in vivo* to mice; however, further systematic investigations are required. Optimization of the surface functionalization to minimize toxic side effects is important. In spite of challenges on the way towards the clinic, carbon nanotubes offer great potential for biomedicine and can bring opportunities for different applications in biomedicine [83–85]. Table 2 summarizes recent carbon nanotube technology for detection of NTs.

## 4. CMOS Technology

Complementary metal-oxide semiconductor (CMOS) technology is a powerful tool for biosensor implementation that is capable of having millions of transistors within system-on-chip and lab-on-chip potentials. Moreover, CMOS has the ability to scale up with tens of nanometers of critical feature size, meeting the requirements for most biosensing schemes [27]. The low-cost mass production of CMOS provides the opportunity to design scalable sensors and provide array implementation for the detection of parallel analytes [91]. In addition, CMOS integrated circuits can be used for signal processing in implantable and portable applications in biological applications [92,93]. Therefore, using CMOS technology in biosensor design could potentially improve the existing sensor performance and open the door to various novel sensing modalities. Scaling up CMOS is not expensive. However, due to the specific materials available in a CMOS process (particularly aluminium and its oxide) as a transducer, this raises the question of the analyte/electrode interface and potential neurotoxicity issues, which is the main drawback of CMOS biosensor commercialisation at present [94]. There are also some limitations in using CMOS technology. The materials used for the fabrication are not biocompatible, so the device must be post-processed. Some techniques have been applied, such as the potentiostatic iodization of the exposed aluminum (Al) in phosphoric acid [95], Ti*/*Pt*/*Au being stacks deposited and structured via lift-off techniques and the plasma-enhanced vapor deposition of silicon oxides and nitrides, followed by platinum (Pt) sputtering to create electrodes [96,97]. In addition, the entire chip, as well as the connections to the external equipment for read out, or driving, or powering of the chip should be protected from the chip, as well. Recently, CMOS technology has been applied for NT monitoring. It provides parallel detection, as well as low-detection limits. Rothe *et al.* have presented an integrated electrochemical measurement system based on CMOS technology that allows the detection of several analytes in parallel (multi-analyte) and that enables simultaneous monitoring at different locations (multi-site). In that study, a label-free, hybridization-based DNA sensor was integrated with CMOS technology.

Furthermore, the detection of the NT, choline, was achieved by assembling the chip with biosensor microprobe arrays [16]. Lu *et al.* have presented miniaturized CMOS capacitive sensors for monitoring DA down to the sub-fM range. The 5×5 sensor array contains five parts composed of various electrode sizes, and each part group's five sensors. No additional lithographic steps were needed after the CMOS foundry process, and CMOS sensor can be used directly [98]. Ayers *et al.* have developed a CMOS potentiostat circuit that can be scaled to a large array [99]; two post-CMOS fabrication methods to incorporate the electrochemical electrode material were presented. The feasibility of on-chip electrochemical measurements of DA and catecholamine release from adrenal chromaffin cells was demonstrated by proof-of-principle. They concluded that the measurement noise was consistent with the typical electrode noise in recordings with external amplifiers. To detect DA, Wang *et al.* also presented interdigitated microelectrodes integrated with CMOS sensing circuitry. The device could be used as an immunosensor, with its sensitivity being enhanced by gold NPs.

**Figure 7.**
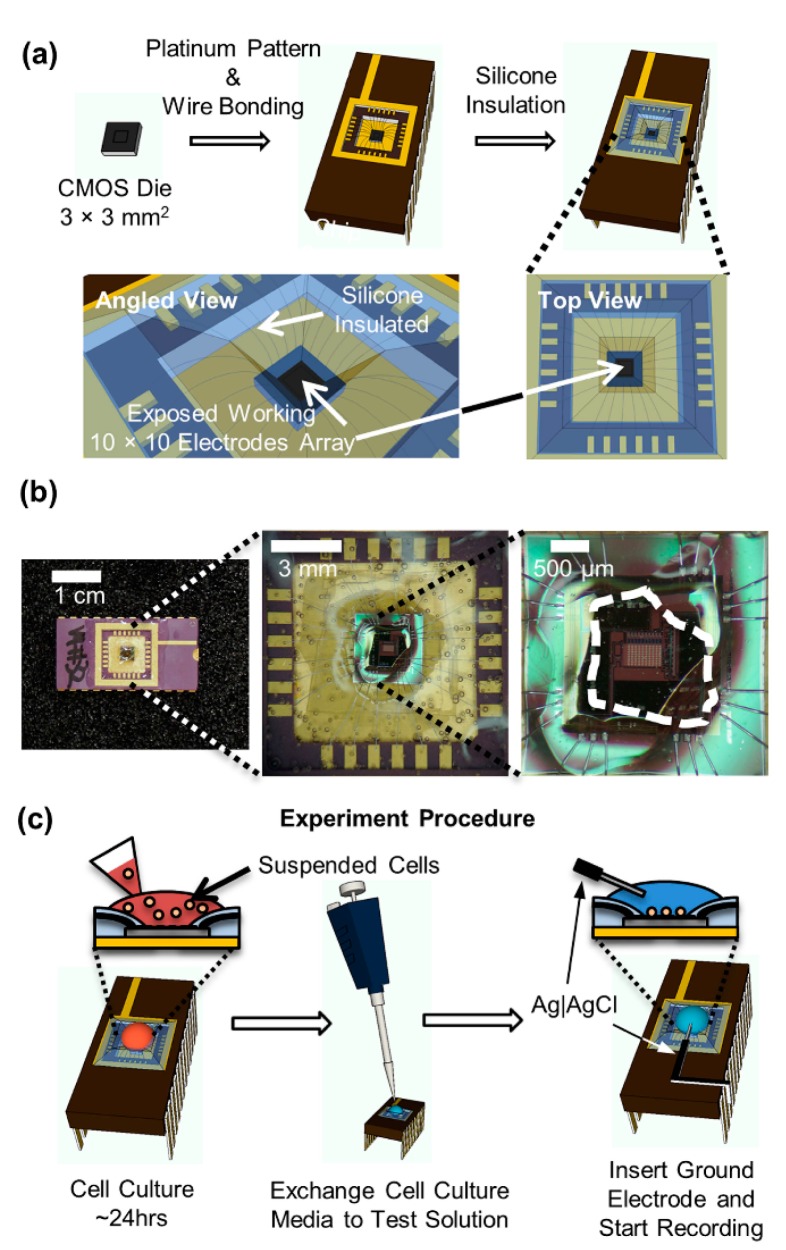
Packaging of the amperometric chip and live-cell experiment procedure. (**a**) A 3 × 3-mm^2^ CMOS die with post-fabricated Pt electrodes was wire bonded to a chip carrier. Silicone was applied at the surface of the chip carrier, insulating the wire bonds and contact pads. This only left the center of the CMOS die exposed where the working electrode array was located; (**b**) Photograph of integrated circuit (IC) biosensor package with silicone insulation of wire bonds and contact pads. The area of the IC that was exposed without silicone coverage is indicated by the white dashed line;(**c**) Schematically shown experiment procedure from the cell culture on the chip to the live cell recording. Reproduced with permission from [101].

Microelectrodes covered by a silicon dioxide layer were fabricated using a maskless post-CMOS process. When charged biomolecules bind to the functionalized oxide surface after immobilization, a capacitive change will be produced for the underlying electrodes [100]. In another work, Kim *et al.* presented an active CMOS-based electrochemical biosensor array with high-throughput capability (100 electrodes) for on-chip amperometric measurement of NT release [101]. The biosensor was designed and fabricated using a combination of CMOS integrated circuit (IC) and a photolithography process to incorporate working platinum electrodes on-chip. They claim that the high throughput of the biosensor array would accelerate the data collection needed to determine the statistical significance of changes produced under changing conditions, from several weeks to a few hours (Figure 7).

Nazari *et al.* [102] have also presented a 16 × 12-channel neurochemical microarray. Each channel acquires bidirectional currents down to pico-amperes, which is proportional to the concentration of a neurochemical. In this study, the on-chip gold microelectrodes were biocompatible, which yielded high sensitivity and selectivity, eliminating costly excessive wiring and minimizing the interference noise. A low-cost on-chip microfluidic network integrated with the CMOS potentiostat array allows for per channel sample delivery and provides shorter analysis and response times and a better overall process control. The microarray enables the simultaneous analysis of up to 192 neurochemicals in parallel.

CMOS technology can also be used for implantable neural prosthetics. Poustinchi *et al.* presented a low-power, noise-immune CMOS-based neurochemical circuit for implantation purposes. Using electrochemical techniques, the device senses picoscale to microscale currents, corresponding to micro-molar NT concentrations. The low-noise characteristics of the device make it suitable for the noisy environment often encountered *in vivo* [103]. The biosensor was designed and fabricated using a combination of CMOS (IC) technology and a photolithography process to incorporate working platinum electrodes on-chip. They claim that the high throughput of the biosensor array will accelerate the data collection needed to determine the statistical significance of changes produced under varying conditions from several weeks to a few hours.

In another study, Goyal *et al.* used an edge plane pyrolytic graphite electrode modified with multi-walled carbon nanotubes (MWNTs/EPPGE) for the quantitative detection of EP in the body fluids of smokers and non-smokers using CV and SWV [77]. The voltammetric method was used to analyze the EP concentration in plasma samples for the first time. Generally, in a natural environment, EP often co-exists with high concentrations of other electroactive biomolecules, such as UA, DA, NE and AA, which interfere with one another. In addition, the product of EP oxidation (EPchrome) can easily be converted into polymers, blocking its further oxidation on the electrode surface. They overcame the UA and AA interference by applying a specific potential region and using MWNT/EPPGE for the determination of catecholamines, thus preventing UA and AA from interfering with the determination [77]. Ly *et al.* investigated the real-time assay of the EP using square-wave anodic stripping voltammetry (SWASV) with a modified carbon nanotube electrode (CNE). They used chelate DNA immobilized on a catalytic CNT surface. The method showed a lower detection limit than other modifications. Thus, it can be attuned to low-detection ranges for *in vivo* analysis and neuro-current detection. A CMOS circuit is also implemented to detect the redox current. Collection efficiency is significantly enhanced via reduced electrode separation [87]. Overall, CMOS technology allows for low-cost, large-scale production of a device to higher densities and larger array sizes for detection of biomolecules, including NTs. Table 3 summarizes recent CMOS techniques for the detection of NTs. Presently, one of the drawbacks for CMOS biosensor commercialisation is the specific materials available in a CMOS process (particularly, aluminium and its oxide), as a transducer raises the problem of the analyte/electrode interface and possible neurotoxicity. To use CMOS as a biosensor, post-processing is required, due to the materials used for the fabrication, which are not biocompatible. Some techniques have been used to overcome these drawbacks, such as potentiostatic iodization of the exposed Al in phosphoric acid, Ti*/*Pt*/*Au stacks deposited and structured via lift-off techniques and plasma-enhanced vapor deposition of silicon oxides and nitrides, followed by sputtering of Pt to make electrodes [94,104,105]. In addition, the whole chip, as well as connections to the external equipment for read out, or to drive, or power the chip should be protected from the chip, as well [16].

## 5. Further Direction

**Figure 8.**
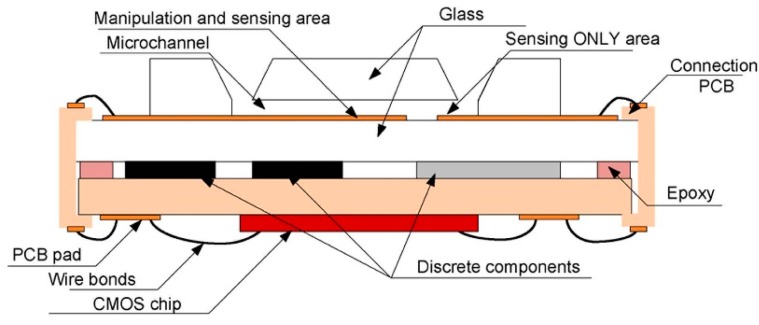
Dielectrophoresis (DEP)-based lab-on-chip (LOC) microsystem architecture using planar electrodes. Reproduced with permission from [109].

To explore new techniques for neurotransmitters detection, we have previously presented a hybrid microelectronics/microfluidic LOC platform; *in vitro* experiments have been performed using artificial cerebrospinal fluid (ACSF) from Tocris Bioscience and microspheres to test the behaviour of the designed LOC [108]. The designed device consists of a fully-integrated dielectrophoresis (DEP)-based LOC with electrodes embedded into a microchannel for sampling brain liquid and injecting it through the microchannel for analysis [109]. Dielectrophoresis **(**DEP) is the movement of particles in a non-uniform electric field, which is due to the interaction of the particles' dipoles and the spatial gradient of the electric field. DEP has been applied to the manipulation of biological particles, such as bacteria, viruses, proteins, nucleic acids and other biomolecules. Its label-free nature, the simplicity of the instrumentation and the ability to induce both negative and positive forces, decrease electrode spacing and increase effective field strengths have been made DEP one of the most popular methods of particle manipulation in microsystems. It is also applicable to non-conducting particles and can be generated either by using a direct current (DC) or alternating current (AC) field. In addition, in the DEP technique, a lower voltage is needed than in electrophoresis, which is suitable for integrated LOC systems. The proposed platform offers a microfluidic system integrated with a microelectronics chip. Both parts are packaged by glass and PDMS followed incorporating into a biocompatible device (Figure 8).

**Figure 9.**
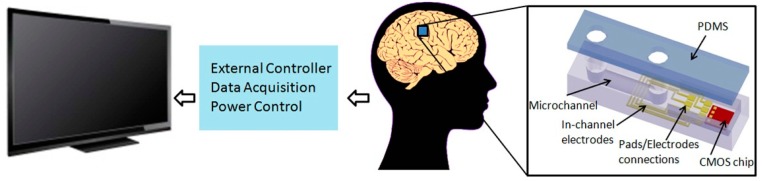
Simplified brain implant device system for the monitoring of several neurotransmitters' concentration variations in real time.

Our team has also recently designed and fabricated a fully-differential complementary metal-oxide semiconductor (CMOS) capacitive sensor and tested the device functionality by using various organic solvents with different dielectric constants [110]. Additionally, we have developed time-based CMOS potentiostats, as well as a multi-electrode amperometric biosensor to detect NTs. The multi-electrode amperometric biosensor can be applied for multi-neurotransmitters detection purposes, which could be valuable to understand many of the human neurological disorders related to NT imbalances (Figure 9) [111].

The integrated potentiostats would also be suitable for the detection circuit of the implantable devices, because of the fast performance of the electrochemical techniques, high sensitivity and temporal resolution, as well as small probe size.

## 6. Applicability

Because the plasma kinetics of most NTs is in nano-molar ranges [112–114], devices with the ability to detect NTs in this range would be applicable. CMOS technology could be a good option for this purpose. As an example, Wang *et al.* designed a CMOS capacitive sensor with sub-μM interdigitated microelectrodes covered by an inter-metal dielectric layer, having a 200 nM limit of detection for DA [100]. In another study, Lu *et al.* designed a 5 × 5 CMOS capacitive sensor array for the detection of DA in fM ranges [98]. It has been shown that, among different architectures for implementing capacitive measurement, charge-based capacitance measurement (CBCM) can achieve unprecedented levels of precision, which enables the measuring of extremely small capacitance changes. Moreover, due to its capability of fabrication in standard CMOS processes, the technique is recognized as a good candidate for use in bio-sensing applications. Additionally, carbon nanotubes have been recently popular for their high sensitivity; with a nanomolar limit of detection for NTs [34,75,89,90]. Because CNT-based sensors have fast electron transfer kinetics, they offer low limits of detection of NTs. Carbon nanofiber electrodes and microelectrodes also offer high sensitivity for NT detection, and the limit of detection has been shown to be in nano-molar ranges [31,48,54,56,66,74]. Carbon nanofiber electrodes and microelectrodes have a small size with the ability to detect rapid changes of potentials, offering nanomolar ranges of NT detection.

## 7. Conclusions

Microelectronic biosensors have the potential to facilitate the development of inexpensive devices for molecular diagnostics. NTs, like most other biomolecules, have extra electrical charges or can be modified with redox-active tags. By applying simple electrochemical detection schemes and using the pre-defined electrical excitations, the monitoring of NTs is possible. This makes microelectronics and microsystems-based platforms useful for NT detection. Microelectronics offer high-performance features in terms of sensitivity, having low background noise, using cost-effective components and being able to perform measurements in turbid samples. In particular, the electrochemical sensor is a completely label-free detection platform, which is beneficial to portable sensors. Microtechnology can also be used as implantable micro-devices with the potential for neuroprosthetic applications, as well as for the investigation of brain circuits *in vivo*. Because of the complications and cost effectiveness of the current devices, research in this area is conducted for simple and cost-efficient devices. In general, micro-devices for *in vivo* and *in vitro* detection of NTs would be promising in the future for the investigation of brain circuits, whereas, in parallel, their potential for neuroprosthetics applications would be valuable.

## Figures and Tables

**Table 1.** Recent voltammetric techniques for detection of neurotransmitters (NTs). DPV, differential pulse voltammetry; FSCV, fast-scan cyclic voltammetry; NE, norepinephrine; WINCS, wireless instantaneous NT concentration system; GC, glassy carbon; CFM, carbon fiber microelectrode; UA, uric acid.

**Sensor**	**Analyte**	**Limit of Detection**	***In Vivo/In Vitro***	**Reference**
Implantable, micromachined microprobe with a microsensor array for combined monitoring of the NTs, GLU and DA, by constant potential amperometry	GLU	2.1 ± 0.2 μM	*In vitro*	[30]
	DA	62 ±8 μM		

Microelectrode, cyclic voltammetry	L-GLU	NA	*In vivo*	[44]

Microelectrode array, DPV, FSCV, amperometry	NE	NA	*In vitro*	[50]

Carbon fiber microelectrodes, Voltammetry	DA, NEP, 5-HT	NA	*In vivo*	[29]

Ultra-microelectrode array, FSCV	DA	NA	*In vitro*	[19]

Thin-film platinum ultramicroelectrode arrays, amperometry	DA	NA	*In vitro*	[51]

Platinum microelectrode, DPV	DA	4.5 nM	*In vivo*	[46]

Enzyme-based microelectrode arrays, amperometry	GLU	0.2 M	*In vitro*	[52]

Carbon-fiber microelectrodes, FSCV	Simultaneous NE-DA	NA	*In vivo*	[53]

Carbon nanofiber electrode, WINCS, FSCV	DA, 5-HT, ascorbic acid	50 nM and 100 nM, respectively	*In vitro*	[54]

High definition cyclic Voltammetry-based on FSCV	DA	NA	*In vitro*	[55]

Molecule-imprinted polyaniline membrane modified on carbon fiber, Amperometry, voltammetry	Glycine	NA	*In vitro*	[56]

Modified GC electrode, CV	DA, UA	2.6 × 10^−7^ M	*In vitro*	[48]
		5.2 × 10^−7^ M		

Microfabricated microelectrodes, FSCV	DA	NA	*In vitro*	[40]

Modified glassy carbon electrode, amperometry	Simultaneous NE, 5-HT	NE: 1.65 × 10^−11^ M	*In vitro*	[57]
		5-HT: 1.32 × 10^−11^ M		

Nanocapillary electrophoretic electrochemical chip, amperometry	DA, NE	30–75 zeptomoles	*In vitro*	[58]

RNA aptamer-based electrochemical biosensor, amperometry	DA	1 μM	*In vitro*	[23]

Capillary zone electrophoresis, amperometry	Six NAAs (neuroactive amino acids): Ala, GLU, Asp, Ser, Tau, Gly, simultaneously	Ranging from 10^−6^ to 10^−7^ mol·L^−1^	*In vitro*	[59]

Amperometry	GLU	NA	*In vitro*	[60]

Chitosan coated CFM, amperometry	5-HT	1.6 nM	*In vivo*	[61]

Selective enzyme immobilization on arrayed microelectrodes, voltammetry	GLU	2.5 ± 1.2 μM	*In vitro*	[62]

Carbon paste electrode modified with cobalt(II) phthalocyanine and tyrosinase, CV	5-HT	0.84 M	*In Vitro*	[63]

Grafting based GLU-AuNPs modified electrode, CV, impedance spectroscopy	NE, UA	1.47 × 10^−10^ M; 1.68 ×10^−11^ M	*In vitro*	[64]

GLU oxidase biosensor based on mixed ceria and titania, NPs, CV	GLU	0.594 M and 0.493 M in oxygenated and deoxygenated conditions	*In vitro*	[65]

Gold nanocluster 2D modified electrode, DPV	DA	0.35–0.51 nM	*In vitro*	[66]

FSCV	NE	NA	*In vivo*	[67]

CE, FSCV	DA, 5-HT, tyramine, octopamine	1, 1, 2.5, 4 nM	*In vivo*	[31]

FSCV	DA, 5-HT, NEP	NA	*In vitro*	[68]

Sandwich- type electrochemical biosensor, phenyl boronic acid immobilized onto gold electrodes, voltammetry	DA	0.2 nM	*In vitro*	[69]

**Table 2.** Recent carbon nanotube technology for detection of NTs. PAR, paracetamol.

**Sensor**	**Analyte**	**Limit of Detection**	***In Vivo/In Vitro***	**Reference**
CNT, Voltammetry	5-HT, DA	Down to sub-μM levels	*In vitro*	[86]

CNT, Voltammetry, chronoamperometry	AD and PAR simultaneously	AD: 0.09 μmol·L^−1^	*In vitro*	[80]
		PAR : 0.06 μmol·L^−1^		

CNT, DPV	DA in urine	0.14 mM	*In vitro*	[24]

CNT electrode, SWASV	EP	1.63 × 10^−9^ M	*In vitro*	[87]

CNT Nanoelectrode Array	GLU	10 nM	10 nM	[74]

CNT, pyrolytic graphite electrode, SWV	EP, NE	0.15 × 10^−9^ and 0: 90 × 10^−10^ M respectively	*In vitro*	[78]

Carbon-fiber microelectrodes coated with Nafion and carbon nanotubes (CNTs)/FSCV	AD	Nafion-CNT electrode: 7± 2 nM; Bare electrode: 21± 3 nM	*In vitro*	[88]

Microelectrodes, CNT, FSCV	DA	17 ± 3 nM	*In vitro*	[73]

CNT, GC, DPV	AA, DA and UA	AA: 0.62 mol·L^−1^	*In vitro*	[79]

CNT, FSCV	5-HT, DA and AA	CONH2-CNT electrodes: 90 nM for 5-HT and 130 nM for DA and for COOH-CNT Modified electrodes: 70 nM for 5-HT and 180 nM for DA	*In vitro*	[75]

Integrated *in vivo* microdialysis electrochemical device, CNT	DA	0.5 nM	*In vivo*	[89]

Thiolated polymeric nanocomposite CNTs DPV	DA	0.4 nmol·L^−1^	*In vitro*	[36]

CNT, FSCV	DA	Bare electrodes: 3.7 ± 0.5 nM oPPY-CNT electrodes: 3.3±0.6 nM Nafion-CNT electrodes: 4±1 nM	*In vivo*	[34]

Ultrasensitive nanowire-transistor	DA	<10^−11^ M	*In vitro*	[90]

**Table 3.** Recent CMOS technology for the detection of NTs.

**Sensor**	**Analyte**	**Limit of Detection**	***In Vivo/In Vitro***	**Reference**
Nafion-coated electrodes, CMOS, CV	DA	0.1 μM	*In vitro*	[106]
5 × 5 CMOS capacitive sensor array	DA	0.1 fM	*In vitro*	[98]
CMOS-based signal processing circuits, carbon nanotube-based sensors	GLU	NA	*In vitro*	[107]
CMOS with sub- μM interdigitated microelectrodes covered by inter-metal dielectric layer	DA	200 nM	*In vitro*	[100]
576 electrode CMOS sensor chip Choline 0.3 M *in vitro* CMOS Potentiostat scalable to large array	Choline	0.3 μM	*In vitro*	[16]
Active CMOS-based electro-chemical biosensor array with high throughput capability (100 electrodes)	DA	0.35 μM	*In vitro*	[101]
